# Meta-analysis of microarray data using a pathway-based approach identifies a 37-gene expression signature for systemic lupus erythematosus in human peripheral blood mononuclear cells

**DOI:** 10.1186/1741-7015-9-65

**Published:** 2011-05-30

**Authors:** Dhivya Arasappan, Weida Tong, Padmaja Mummaneni, Hong Fang, Shashi Amur

**Affiliations:** 1 National Center for Toxicological Research, US Food and Drug Administration, 3900 NCTR Road, Jefferson, AR 72079, USA; 2 Genomics Group, Office of Clinical Pharmacology, Office of Translational Science, Center for Drug Evaluation and Research, US Food and Drug Administration, 10903 New Hampshire Avenue, Silver Spring, MD 20993, USA; 3 Z-Tech Corporation, an ICF International Company at the National Center for Toxicological Research, US Food and Drug Administration, 3900 NCTR Road, HFT 230, Jefferson, AR 72079, USA; 4 University of Texas, Austin, Texas Institute for Drug and Diagnostic Development, 2500 Speedway, TX 78712, USA

## Abstract

**Background:**

A number of publications have reported the use of microarray technology to identify gene expression signatures to infer mechanisms and pathways associated with systemic lupus erythematosus (SLE) in human peripheral blood mononuclear cells. However, meta-analysis approaches with microarray data have not been well-explored in SLE.

**Methods:**

In this study, a pathway-based meta-analysis was applied to four independent gene expression oligonucleotide microarray data sets to identify gene expression signatures for SLE, and these data sets were confirmed by a fifth independent data set.

**Results:**

Differentially expressed genes (DEGs) were identified in each data set by comparing expression microarray data from control samples and SLE samples. Using Ingenuity Pathway Analysis software, pathways associated with the DEGs were identified in each of the four data sets. Using the leave one data set out pathway-based meta-analysis approach, a 37-gene metasignature was identified. This SLE metasignature clearly distinguished SLE patients from controls as observed by unsupervised learning methods. The final confirmation of the metasignature was achieved by applying the metasignature to a fifth independent data set.

**Conclusions:**

The novel pathway-based meta-analysis approach proved to be a useful technique for grouping disparate microarray data sets. This technique allowed for validated conclusions to be drawn across four different data sets and confirmed by an independent fifth data set. The metasignature and pathways identified by using this approach may serve as a source for identifying therapeutic targets for SLE and may possibly be used for diagnostic and monitoring purposes. Moreover, the meta-analysis approach provides a simple, intuitive solution for combining disparate microarray data sets to identify a strong metasignature.

Please see Research Highlight: http://genomemedicine.com/content/3/5/30

## Background

Microarrays are powerful tools with capability of measuring the transcript abundance of tens of thousands of genes simultaneously in biological samples. Microarray technology has matured over the past 15 years and is now employed for the study of gene expression signatures associated with disease [1-3]. The clinical utility of microarrays as prognostic tools can be evidenced by the approval of the US Food and Drug Administration (FDA) of a customized microarray, MammaPrint™ (Agendia, Amsterdam, The Netherlands) for predicting the outcomes in breast cancer patients on the basis of a 70-gene expression signature [4].

Some of the challenges associated with identification of gene expression signatures that differentiate the disease state from healthy controls are the availability of samples, sample size, heterogeneous data sets, and reproducibility. Thus, robustness of the gene expression signature derived from one study needs to be validated by other independent studies, preferably with large sample sizes. In practice, however, several studies with relatively small sample sizes are often used to identify gene expression signatures. In these circumstances, it is beneficial to combine the results of several individual studies using meta-analysis. This process enhances statistical power in identifying more robust and reliable gene signatures.

Several meta-analysis approaches have been proposed specifically for handling heterogeneous data sets. For example, Rhodes *et al. *[5] used the approach of utilizing *P *values of genes across studies to identify gene expression signatures that differentiate cancer tissues from normal tissues and to predict poor or good patient outcomes. Choi *et al. *[6] used an effect size estimate approach in a meta-analysis of two cDNA microarray data sets, human hepatocellular carcinoma and prostate cancer, to identify a transcriptional signature for cancer. A Bayesian approach was used by Wang *et al. *[7], who performed microarray studies on three different platforms and combined them to study differences in gene expression between B-cell chronic lymphocytic leukemia and normal B cells. Shen *et al. *[8] suggested a Bayesian mixture model incorporating the probability of expression measure.

**Figure 1:**
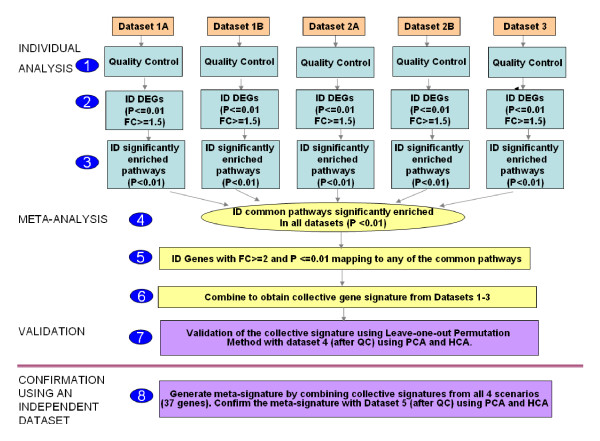
**Pathway based meta-analysis process (described for scenario I in Tables 2 and 3)**. The meta-analysis approach involved three major steps: individual analysis of the data sets, meta-analysis at the pathway level, and validation of the signature. Figure 1 represents the process for one scenario. For each scenario, three of the data sets were used to generate the signature and the fourth one was used for testing of the signature. The four data sets were switched around to create four scenarios (see Table 2). The signatures from each scenario were then combined to provide a meta-signature, which was confirmed by the fifth data set.

Most of the currently used meta-analysis approaches first identify a set of commonly probed genes across studies and then derive a gene expression signature from these. A shortcoming of this approach is a potential loss of valuable information from individual data sets during the combining process. Thus, we propose a pathway-based meta-analysis approach whereby differentially expressed genes (DEGs) from individual studies are selected using a combination of *P *value and fold change and the results are combined at the pathway level instead of at the gene level (see Figure 1 and Methods). Additionally, while most other methods perform very little validation or rely solely on the biological plausibility of the obtained results to serve as validation, the approach proposed here includes statistical validation through the leave one data set out permutation method. The results are further confirmed using an independent data set.

A number of authors have reported the use of microarray technology to identify gene expression signatures in systemic lupus erythematosus (SLE) [9-14], mechanisms underpinning SLE [15-17], and pathways related to SLE [18-20]. However, meta-analysis approaches have not been explored sufficiently in the study of SLE microarray data [21]. In the present study, the pathway-based meta-analysis method was applied to four independent gene expression oligonucleotide microarray data sets to identify gene expression signatures for SLE. These data sets were generated from peripheral blood mononuclear cell (PBMC) samples from SLE patients and healthy controls. The resulting signatures were then confirmed by testing on a fifth independent data set.

## Methods

### Data collection

Data sets from five independent microarray studies comparing PBMC samples from SLE patients with those from healthy individuals were obtained from prominent SLE researchers. These data sets are referred to as data sets 1, 2, 3, 4, and 5. Data sets 1, 2, 4, and 5 are associated with peer-reviewed publications (11-14). Data set 3 is composed of unpublished data. Three of the studies (studies 1, 4, and 5) included only pediatric patients, while the remaining two included only adults. All studies employed the Affymetrix GeneChipmicroarray platform (Affymetrix, Inc., Santa Clara, CA, USA) but the versions of the array type varied (Table 1). In the case of two different array types used for the same study (that is, data sets 1 and 2), we treated them as separate data sets (data sets 1a, 1b, 2a, and 2b) during the meta-analysis. Raw data in the form of Affymetrix CEL files were provided for studies 1, 2, 3, and 5. For data set 4, however, expression values for a short-listed set of genes were provided. While data sets 1 to 4 were used in the meta-analysis workflow, data set 5 served as an independent data set to validate the gene signature derived from the meta-analysis.

**Table 1:** Information on data sets used for meta-analysis^a^

Data set	Platform	Type of data	Samples, *n*	Source
Data set 1	1a: HG U133A 1b: HG U133B	Pediatric PBMC	59 (38 SLE, 21 controls)	Allantaz *et al. *[11]
Data set 2	2a: HG U95 Av1 2b: HG U95 Av2	Adult PBMC	90 (48 SLE, 42 controls)	Baechler *et al. *[12]
Data set 3	HG U133 Plus 2	Adult PBMC	58 (44 SLE, 14 controls)	Unpublished data
Data set 4	HG U95 Av1	Pediatric PBMC	39 (30 SLE, 9 controls)	Bennett *et al. *[35]
Data set 5	1a: HG U133A 1b: HG U133B	Pediatric PBMC	57 (47 SLE, 10 controls)	Chaussabel *et al. *[14]

^a^PBMC, peripheral blood mononuclear cell; SLE, systemic lupus erythematosus.

### Workflow of the pathway-based meta-analysis approach

The overall workflow of the pathway-based meta-analysis is summarized in Figure 1. The meta-analysis used a leave one data set out validation process. Both principal component analysis (PCA) and hierarchical cluster analysis (HCA) were used to visually inspect the leave one data set out cross-validation results. Last, the combined meta-signature obtained from the 4 data sets was validated against an independent fifth data set (data set 5).

For the individual quality control and data analysis steps mentioned below, each data set was considered separately. Additionally, since data sets 1 and 2 used two chip types each, they were considered as four different data sets (1a, 1b, 2a, and 2b) for the initial analysis.

### Quality control

Quality assessment was done for each data set using the Genedata Expressionist (Genedata, San Francisco, CA, USA) [22] (Figure 1, step 1). Only one sample in data set 2a was discarded from further analysis, because it had too high a value for defective area percentage.

### Individual data processing and analysis

Following quality control assessment, each data set was analyzed individually using the ArrayTrack™ tool (US Food and Drug Administration's National Center for Toxicological Research, Jefferson, AR, USA) [23]. ArrayTrack is a comprehensive tool for microarray data storage, analysis, and interpretation that has been developed at the FDA's National Center for Toxicological Research. To maintain consistency during the individual analysis of data sets, similar normalization methods, statistical tests, and parameters were used with all data sets. First, all data sets except data set 4 were normalized using Robust Multi-array Analysis. Then Welch's *t *test was performed on each data set individually. The *P *value and fold change filters (0.01 and 1.5, respectively) were used to identify a unique list of DEGs from each data set (Figure 1, step 2). This list represented genes that were either notably upregulated or downregulated in the PBMCs of SLE patients when compared to the PBMCs of healthy controls. Each DEG list was then used to identify biological pathways significantly represented in SLE samples compared to the healthy controls (*P *< 0.01) in each data set (Figure 1, step 3). This pathway analysis was done using Ingenuity Pathway Analysis (IPA) software (Ingenuity Systems Inc., Redwood City, CA, USA).

### Meta-analysis

Pathways common to all of the data sets were identified from the individual lists of pathways enriched in SLE patients compared to healthy controls (one for each data set) (Figure 1, step 4). The resulting list of pathways was indicative of processes significantly affected in all of the SLE data sets and comprised a pathway signature representative of all data sets and of the disease. From this common pathway signature, gene markers that met all of the following criteria were selected: (1) exhibited a fold change greater than 2 in at least one of the data sets (stringency increased from 1.5-fold to 2-fold to obtain a robust signature), (2) present in the DEG list in at least one of the data sets, and (3) involved in at least one of the commonly enriched pathways (Figure 1, step 5). These DEGs composed the collective signature (Figure 1, step 6).

### Validation with the leave one data set out permutation method

**Figure 2:**
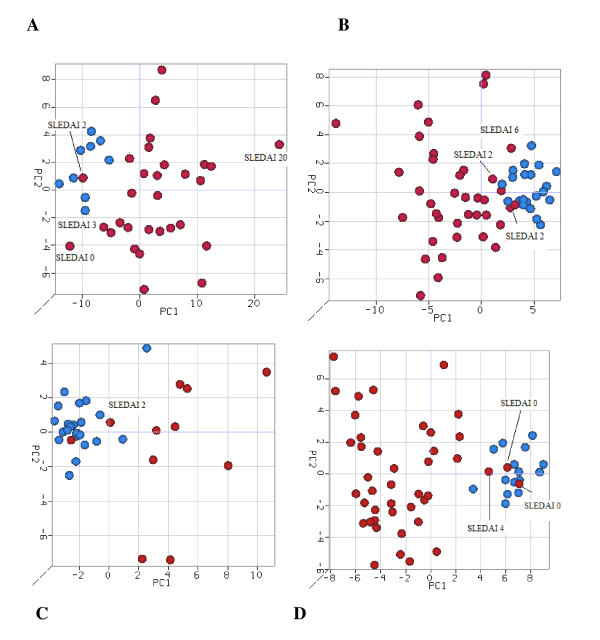
**Principal component analysis from all scenarios**. There is a clear distinction between healthy samples and systemic lupus erythematosus (SLE) patients, shown in blue and red, respectively. **(A) **Scenario I. **(B) **Scenario II. **(C) **Scenario III. **(D) **Scenario IV. The Systemic Lupus Erythematosus Disease Activity Index (SLEDAI) scores indicate that most SLE patients exhibiting close to normal gene expression are in remission (SLEDAI score 0) or have low disease activity (SLEDAI scores 2 and 3).

**Figure 3:**
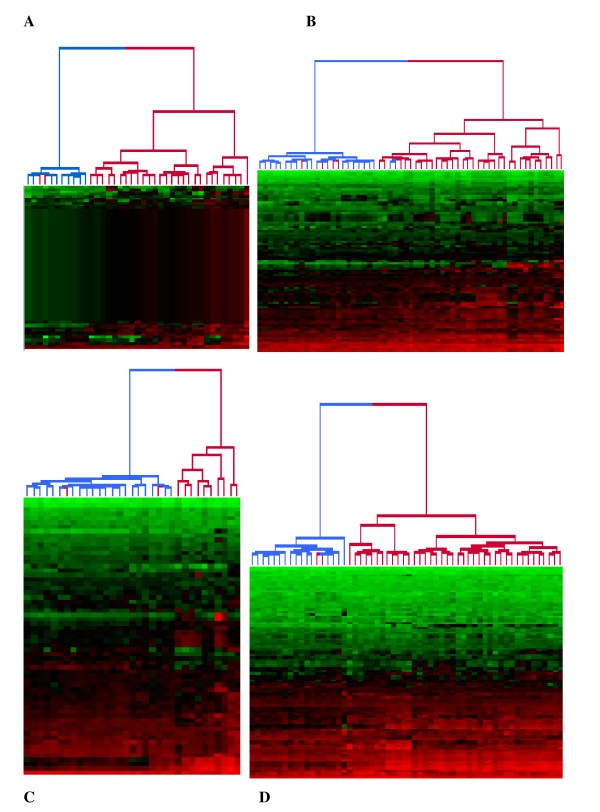
**Hierarchical clustering analysis for all scenarios**. Blue branches indicate healthy samples, and red branches indicate SLE patients. **(A) **Scenario I. **(B) **Scenario II. **(C) **Scenario III. **(D) **Scenario IV.

To validate this technique, a leave one data set out permutation approach was employed (Figure 1, step 7). The meta-analysis technique described above was reiterated four times, each time leaving out one of the four data sets (data sets 1 to 4) and performing the analysis using the remaining three data sets. This gave rise to four different scenarios (Table 2). The gene signature obtained using the three data sets (for example, data sets 1 to 3) was then applied to the data set left out (for example, data set 4). Unsupervised visualization techniques such as PCA and HCA were performed to examine how well the signature could differentiate SLE patients from healthy controls (Figures 2 and 3).

**Table 2:** Scenarios for leave one data set out validation

Scenario I	Scenario II	Scenario III	Scenario IV
Data set 1 (a and b)	Data set 2 (a and b)	Data set 1 (a and b)	Data set 1 (a and b)
Data set 2 (a and b)	Data set 3	Data set 3	Data set 2 (a and b)
Data set 3	Data set 4	Data set 4	Data set 4
Test: Data set 4	Test: Data set 1 (a and b)	Test: Data set 2 (a and b)	Test: Data set 3

### Metasignature development

Gene markers were generated for each of the four scenarios as described in the Meta-analysis section. Gene markers present in at least three of the four scenarios were grouped to comprise a 37-gene metasignature.

### Confirmation using a fifth independent data set

**Figure 4:**
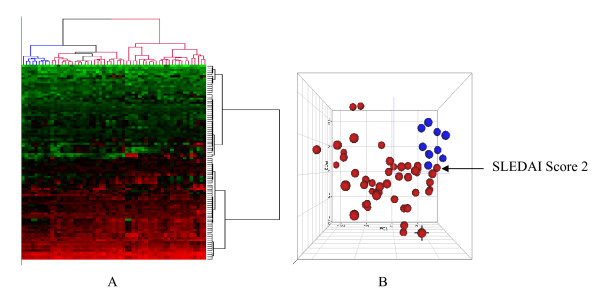
**Validating the 37-gene signature using independent data set 5**. **(A) **Hierarchical clustering analysis shows blue branches indicating healthy samples and red branches indicating SLE patients. **(B) **Principal component analysis with healthy samples shown in blue and SLE patients shown in red.

Confirmation of the final 37-gene metasignature was done using an independent fifth data set, data set 5 (Figure 1, step 8). Again, PCA and HCA were carried out to evaluate the ability of the metasignature to differentiate SLE patients from healthy controls in this independent data set (Figure 4).

### Results and discussion

Individual data sets of SLE and healthy control data sets derived from Affymetrix microarrays were analyzed using ArrayTrack following quality control (Figure 1, step 1) and normalization procedures. DEGs for individual data sets were identified using a *P *value cutoff of 0.01 and a fold change cutoff of 1.5 (Figure 1, step 2).

### Biological pathways identified in SLE patients through the leave one data set out permutation method

After applying the leave one data set out approach for each of the four scenarios (Table 2), commonly enriched biological pathways were identified using IPA software (Table 3). Three biological pathways were consistently enriched in SLE patients in all four scenarios: interferon (IFN) signaling, interleukin (IL)-10 signaling, and glucocorticoid receptor signaling. An additional pathway, LXR/RXR signaling, was identified only in scenario IV.

**Table 3:** Biological pathways commonly and significantly enriched in the four scenarios^a^

Scenario I	Scenario II	Scenario III	Scenario IV
IPA pathways	IPA pathways	IPA pathways	IPA pathways
IL-10 signaling	IL-10 signaling	IL-10 signaling	IL-10 signaling
Interferon signaling	Interferon signaling	Interferon signaling	Interferon signaling
Glucocorticoid receptor signaling	Glucocorticoid receptor signaling	Glucocorticoid receptor signaling	Glucocorticoid receptor signaling
			LXR/RXR signaling

^a^IPA, Ingenuity Pathway Analysis software; IL, interleukin; LXR, liver X receptor; RXR, retinoid X receptor.

Previous studies have provided evidence of increased autoimmunity in patients undergoing IFN treatment [24]. More specifically, there is evidence of women developing SLE during IFN-α treatment [25]. Several studies have shown upregulation of the IFN signaling pathway in SLE patients [9,12,17,26-30]. Therefore, it is understandable that IFN signaling appears to be affected across all data sets.

IL-10 signaling appears to be dysregulated and may be indicative of the inflammatory processes involved in SLE. IL-10 binds to IL-10 receptor 1 on immune cells and activates the JAK-STAT signaling pathway, which is the key IFN signaling mechanism [31]. In support of the hypothesis that IL-10 is involved in SLE, IL-10 has been identified as one of risk loci for SLE in a large genome-wide association study [32].

Glucocorticoid receptors are also believed to influence cytokine signaling and may be indirectly involved in the pathways underpinning SLE [33]. In fact, glucocorticoids are routinely used in the treatment of SLE patients.

### Genes differentially expressed in SLE

Each of the four scenarios produced a gene signature: scenario I produced a signature comprising 51 genes, scenario II produced a signature with 31 genes, scenario III produced a signature with 34 genes, and scenario IV produced a signature with 28 genes. These DEGs represent the three main SLE disease pathways (IFN signaling, IL-10 signaling pathway, and glucocorticoid signaling pathways) as discussed in the section above.

A separate analysis of the pediatric and adult data sets used to identify DEGs and pathways in the two populations was conducted. Similar gene expression patterns were observed in adult and pediatric populations, although the extent of upregulation of some of the genes was higher in the pediatric data sets (unpublished results).

### Validation of the meta-analysis approach

For each scenario, the signature obtained using the three data sets was applied to the fourth data set to observe how effectively the expression of the signature genes could distinguish between the SLE and healthy populations.

The PCAs and HCAs obtained for each scenario are presented in Figures 2 and 3, respectively. The PCA and HCA produced similar and consistent results. Grouping of samples based on the expression of signature genes alone produced a clear distinction between SLE patients and healthy controls. The results suggest that the DEG signatures derived by using the leave one data set out permutation approach in the four scenarios (Table 2) can potentially identify a robust gene expression signature for SLE.

### Gene expression signatures for SLE and Systemic Lupus Erythematosus Disease Activity Index scores

The Systemic Lupus Erythematosus Disease Activity Index (SLEDAI) is a validated scoring system that can be used to describe the range of disease activity and comprises a weighted score calculated by the presence or absence of 24 symptoms. The association of SLEDAI scores to expression profiles of SLE patients was evaluated. While the majority of the samples were grouped into their respective classes (SLE or control; see Figure 2), 12 SLE patients exhibited expression profiles similar to the control samples. On closer examination of these samples, it was found that the scores for nine of the patients indicated that they were either in remission (SLEDAI score 0) or had mild activity of the disease (SLEDAI score 2 or 3). These findings lend further credence to the ability of the pathway-based meta-analysis approach used here in distinguishing SLE patients from healthy controls. Correlation between SLEDAI scores and gene expression signatures has also been reported in the literature [9,26,34].

### Metasignature for SLE

**Figure 5:**
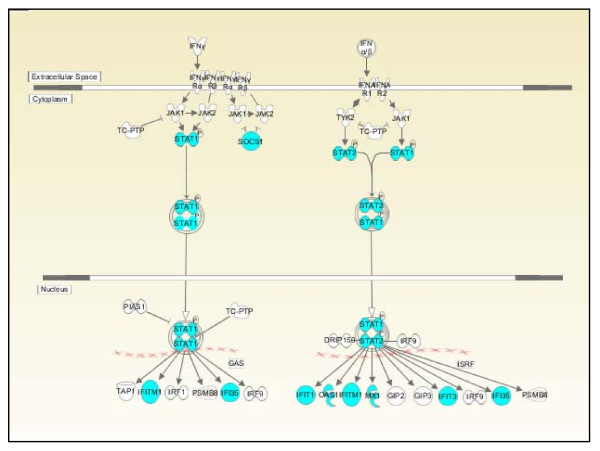
**Interferon signaling pathways**. Interferon-α, interferon-β, and interferon-γ signaling pathways are shown. The genes in blue represent differentially expressed genes that are part of the SLE metasignature.

A 37-gene signature was generated by the meta-analysis workflow (Table 4). Many IFN-induced genes involved in the IFN signaling pathway (Figure 5), such as *IFIT1, IFIT3, IFITM1, IFIT35, MX1*, and *OAS1*, were present in the signature. Overexpression of IFN-regulated genes in PBMCs of SLE patients has been reported in several publications [9,15,26,35-37]. In addition to genes involved in the IFN signaling pathway, genes in cytokine signal transduction (*SOCS1 *and *SOCS3*) were also among the DEGs in SLE patients. Differential expression of many other biomarkers associated with inflammatory and/or immune responses and with cellular proliferation was also observed, as shown in Table 4.

**Table 4:** Signature genes and their functionalities

Functionality	Genes from signature
Interferon signaling and interferon-induced or interferon-regulated genes	*IFI35, IFIT1, IFIT3, IFITM1, MX1, OAS1, STAT1, STAT2, CCL2, CXCL1, SOCS1, SOCS3*
Inflammatory and immune response	*CCL3, CCR1, CD163, FCGR1A, IL1R2, IL1B, IL1RN, IL-8, NFAT5, TRA@, MAP2K3, MAP2K6, SMAD3, FCGR2A, FCGR2B, NFKBIA*
Cellular proliferation and differentiation	*CDKN1C, CDK1A, DUSP1, EP300, FOS, JUN, PRKACA, PRKACB*
Protein folding	*SLP1*

### Confirmation of the metasignature using an independent data set

This signature was applied to an independent fifth data set (data set 5) to evaluate its ability to distinguish the SLE samples from the control samples. Figure 4 shows that the signature demonstrated clear differentiation between SLE patients and healthy controls. In the HCA analysis, nine of ten healthy samples clustered together and were clearly separated from the cluster of SLE samples (Figure 4A). The PCA analysis also showed that the majority of the SLE samples and healthy samples were grouped separately (Figure 4B). The one SLE sample that was clustered with the healthy samples had a SLEDAI score of 2, confirming our earlier observations with different data sets (Figure 2).

## Conclusions

The novel pathway-based meta-analysis approach proved to be a useful technique for grouping disparate microarray data sets. This technique allowed for validated conclusions to be drawn across four different data sets and confirmed by testing on an independent fifth data set. Since the metasignature was obtained from pathways that were enriched in SLE samples across all of the data sets, it is highly representative of biological pathways related to SLE. The metasignature produced here may serve as a source for identifying potential therapeutic targets for SLE. Also, with further refinement, it might be made clinically more intuitive, which may also prove to be useful for the diagnosis and monitoring of SLE. Moreover, the meta-analysis approach outlined here provides a simple and intuitive solution for combining disparate microarray data sets to identify a robust metasignature.

## Competing interests

The authors declare that they have no competing interests.

## Authors' contributions

DA created the first draft of the manuscript and performed the meta-analysis. PM and SA performed the analysis to confirm the metasignature in an independent data set. SA, PM, and HF helped significantly to draft the manuscript. SA and WT helped coordinate the project and finalize the manuscript. All authors read and approved the final manuscript.

## Pre-publication history

The pre-publication history for this paper can be accessed here:

http://www.biomedcentral.com/1741-7015/9/65/prepub
